# Longitudinal Surveillance of COVID-19 Antibodies in Pediatric Healthcare Workers

**DOI:** 10.3390/vaccines13020163

**Published:** 2025-02-07

**Authors:** Dunia Hatabah, Sneh Lata Gupta, Grace Mantus, Patrick Sullivan, Stacy Heilman, Andres Camacho-Gonzalez, Deborah Leake, Mimi Le, Mark Griffiths, Carson Norwood, Samuel Shih, Rawan Korman, Giorgi Maziashvili, Chris A. Rees, Laura Benedit, Bridget A. Wynn, Mehul Suthar, Miriam B. Vos, Jens Wrammert, Claudia R. Morris

**Affiliations:** 1 Department of Pediatrics, Emory University School of Medicine, Atlanta, GA 30322, USA; dunia.hatabah@emory.edu (D.H.); snehlata@med.umich.edu (S.L.G.); grace.mantus@nih.gov (G.M.); sheilma@emory.edu (S.H.); acamac2@emory.edu (A.C.-G.); uyen.quynh.nguyen.le@emory.edu (M.L.); mark.anthony.griffiths@emory.edu (M.G.); carsonenorwood@gmail.com (C.N.); samuelatshih@gmail.com (S.S.); rawan.korman@emory.edu (R.K.); giorgi.maziashvili@emory.edu (G.M.); chris.rees@emory.edu (C.A.R.); bridget.wynn@emory.edu (B.A.W.); mehul.s.suthar@emory.edu (M.S.); mvos@emory.edu (M.B.V.); jwramme@emory.edu (J.W.); 2 Center for Childhood Infections and Vaccines of Children’s Healthcare of Atlanta and Emory University, Atlanta, GA 30322, USA; 3 Department of Epidemiology, Rollins School of Public Health, Emory University, Atlanta, GA 30329, USA; pssulli@emory.edu; 4 Children’s Healthcare of Atlanta, Atlanta, GA 30329, USA; deborah.leake@choa.org (D.L.); laura.benedit@choa.edu (L.B.); 5 Center for Clinical and Translational Research of Children’s Healthcare of Atlanta and Emory University, Atlanta, GA 30322, USA

**Keywords:** COVID-19, SARS-CoV-2, spike, pediatric healthcare workers, COVID-19 vaccine, seasonal coronaviruses, booster

## Abstract

**Background:** Vaccines against COVID-19 target the spike protein. There is minimal information on longitudinal COVID-19 immune profiling in recovered versus naïve and vaccinated versus non-vaccinated healthcare workers (HCWs). **Methods:** This is a prospective longitudinal observational cohort of pediatric HCWs (pHCWs) conducted during 2020–2022 at an academic center, exploring the impact of COVID-19 vaccination on immunoglobulin G (IgG) antibody titers over time and cross-reactivity with other coronaviruses, including SARS-CoV-1, MERS-CoV, and seasonal coronaviruses (HCoV-HKU1 and HCoV-OC43). **Results:** A total of 642 pHCWs initially enrolled, and 337 participants had repeat IgG titers measured post-vaccine and post-booster. Most participants were female, median age range of 31–40 years. Anti-spike was higher in all vaccinated individuals versus non-vaccinated (*p* < 0.0001) and naïve versus infected (*p* < 0.0001). A single dose of vaccine was sufficient to attain maximum titers in recovered participants versus naïve who received both doses of vaccine. Anti-spike titers dropped significantly at 9 months after the primary series, whereas sustained anti-spike titers were observed at 9 months post-booster. **Conclusions:** All vaccinated pHCWs developed antibodies to spike. COVID-19 infection and/or vaccination yielded antibodies that cross-reacted to SARS-CoV-1, MERS-CoV, HCoV-HKU1, and HCoV-OC43. Anti-spike titers were more durable post-booster compared to the primary series. Longitudinal immune profiling of COVID-19 responses provides vital data to shape public health policies, optimize vaccine strategies, and strengthen pandemic preparedness.

## 1. Introduction

Severe Acute Respiratory Syndrome Coronavirus 2 (SARS-CoV-2) is the causative agent of COVID-19 that has been linked to more than seven million deaths worldwide [1]. Control of the COVID-19 pandemic was, in part, achieved through mass vaccination against SARS-CoV-2. In December 2020 the Food and Drug Administration issued emergency use authorization for the mRNA vaccines BNT162b2 (Pfizer–BioNTech) and mRNA-1273 (Moderna) [2]. Both mRNA vaccines target the spike protein that is shared by several coronaviruses, which may impart partial immunity to coronaviruses previously identified. There is minimal information on longitudinal immune profiling of different sub-groups, including COVID-19-recovered (i.e., those who were previously infected) vs. naïve (i.e., those who were never infected) and vaccinated vs. non-vaccinated individuals.

Healthcare workers (HCWs) served as the frontline working force during the COVID-19 pandemic, and vaccination for HCWs in the United States started in December 2020. The pediatric healthcare workforce has been an understudied professional category affected by the pandemic, because children were thought to be at a lower risk of infection, suggesting minimal risk of work-acquired COVID-19 infection in this professional category early in the pandemic [3]. It is now established that children may have similar or even higher rates of COVID-19 infection but, are more frequently asymptomatic [4,5,6]. Hence, it is possible that children infected with COVID-19 may have put frontline pediatric healthcare workers (pHCWs) at higher risk of COVID-19 infection, particularly early in the pandemic. Children have higher rates of common cold infections than adults, including infections with endemic human coronaviruses (HCoV) HKU1 and OC43 [7,8]. Children have been shown to generate robust antibodies against HCoVs after SARS-CoV-2 infection, indicating cross-reactivity, the benefit of which is yet to be explored [9,10,11]. Given their interaction with children, pHCWs also have higher rates of exposure to coronaviruses that cause the common cold than the general population.

There remains little clarity regarding antibody titers among HCWs according to specific clinical settings; hence, it is unknown whether HCW-specific clinical characteristics and outcomes differ from the general population due to repeated exposure to the virus and other viruses in general. We studied a cohort of pHCWs investigating the epidemiology of COVID-19 infection, a professional category that has been underrepresented in the literature. We have previously shown that the prevalence of COVID-19 in pHCWs was 4.1% and that pHCWs working in the emergency department had a four-fold higher prevalence of infection before universal masking policies were in place, highlighting job location as a possible risk factor for COVID-19 infection [12]. In a subsequent analysis, we showed an incidence of 8.2% in seropositivity in the same cohort, that was no longer job-specific, highlighting the possible role of universal adoption of personal protective equipment (PPE) in mitigating the initial risk of exposure experienced by frontline pHCWs [13]. The use of PPE has been shown to potentially protect against infection in another cohort of pHCWs [14]. In addition, we showed that antibody titers to COVID-19 decline rapidly over time, and titers against the receptor-binding domain (RBD) of SARS-CoV-2 correlated strongly with neutralizing antibodies [13]. This correlation has been supported in the literature and suggests that a stronger antibody response to RBD will likely have more protection against COVID-19 infection [15]. Here, we monitor serological antibody responses elicited by COVID-19 infection and vaccination in a prospective cohort of pHCWs within a year post-vaccination initiation.

## 2. Materials and Methods

### 2.1. Study Design

**Figure 1:**
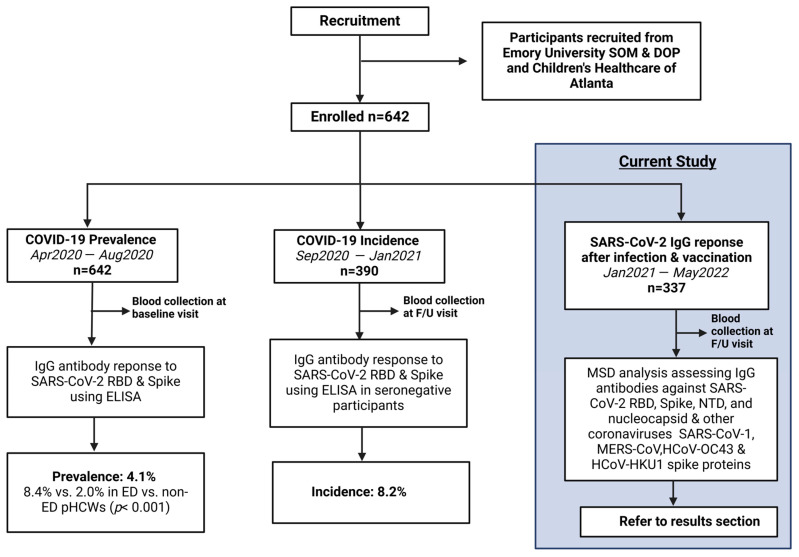
Study Flow Diagram: Overall study procedures across the three different stages of analysis of COVID-19 epidemiology in a cohort of pediatric healthcare workers. From April 2020 to August 2020, 642 pHCWs were assessed at baseline for IgG antibody response to SARS-CoV-2 RBD and spike utilizing ELISA, showing a prevalence of 4.1% that was significantly higher in emergency department pHCWs. From September 2020 to January 2021, 390 seronegative pHCWs returning for at least 1 follow-up (F/U) visit were assessed for incidence of new seropositivity using ELISA, showing a new incidence of 8.2%. The current study was conducted from January 2021 to May 2022, enrolling 337 participants from the original cohort who returned for at least one follow-up visit post-vaccination availability, conducting MSD analysis assessing antibodies against SARS-CoV-2 antigens and other coronaviruses.

This is an analysis of an ongoing prospective longitudinal cohort study with cross-sectional analyses investigating the epidemiology of COVID-19 in pHCWs, conducted from April 2020 through May 2022. The cohort consisted of pHCWs who provided medical care in the largest pediatric healthcare system in Georgia, caring annually for over 250,000 infants, children, and adolescents across 3 pediatric emergency departments (ED). Baseline prevalence and incidence results were previously published [12,13]. Subsequently, a cohort of returning pHCWs was assessed for immunoglobulin (IgG) titer levels within 3 months following the primary series of COVID-19 vaccination and again after booster from January 2021 to May 2022 (Figure 1).

### 2.2. Participants

Participants were recruited from Emory University School of Medicine Department of Pediatrics (DOP), Children’s Healthcare of Atlanta, and private pediatric practitioners or contractors who worked regularly at Children’s Healthcare of Atlanta facilities. This included healthcare employees and support staff, who were recruited via email, physical postings in the various Children’s Healthcare of Atlanta and DOP locations, as well as by personal communication. The study did not enroll acutely symptomatic participants or participants with a history of blood clotting disorders. There were no other exclusion criteria, and all pHCWs within the pediatric healthcare network were welcome to participate. To best ensure research staff safety, all participants were required to be asymptomatic at the time of in-person visits. Participants were told in advance that if they were symptomatic near the time of participation, they would be rescheduled for a later date that was at least 14 days post symptoms onsets. Participants were categorized based on vaccination and infection status: Recovered Vaccinated, Naïve Vaccinated, Recovered Unvaccinated, and Naïve Unvaccinated.

### 2.3. Procedures

Informed consent was obtained from each participant via verbal or electronic format, and each participant completed an online survey verifying demographic and occupational information, such as age range by decade, sex, and history of comorbidity. In addition, each participant reported incident infections during follow-up visit(s). Participants were asked to return for follow-up visits every 3 months for the duration of the pandemic, or sooner if they were diagnosed with COVID-19 and subsequently recovered. All participants provided up to 30 mL of blood via venipuncture. Blood specimens were processed within 1 h of collection, and serum and plasma were stored by the Children’s Healthcare of Atlanta, Emory University, and the Children’s Healthcare of Atlanta Clinical and Translational Discovery Core at –20 °C for future analysis.

### 2.4. Laboratory Methods

Serum samples were analyzed using a multiplex electro-chemiluminescent-based multiplex immunoassay provided by Mesoscale Discovery (MSD; Rockville, MD 20850-3173, USA). The assay was performed according to the manufacturer’s instructions using the V-PLEX COVID-19 Coronavirus Panel 1 (K15362U). This panel contains antigens for wild-type (Wuhan strain) SARS-CoV-2 receptor-binding domain (RBD), Spike, N-terminal domain (NTD), and nucleocapsid proteins, along with other beta coronaviruses, such as Severe Acute Respiratory Syndrome Coronavirus 1 (SARS-CoV-1), Middle East Respiratory Syndrome Coronavirus (MERS-CoV), Human Coronavirus OC43 (HCoV-OC43), and Human Coronavirus HKU1 (HCoV-HKU1) spike proteins. Briefly, serum samples were diluted 1:5000 prior to the assay. Kit plates were blocked for 30 min using the manufacturer-provided blocking buffer A. The blocking buffer was removed, and the plates were washed 3 times with a wash buffer. Diluted serum samples and Mesoscale standard controls were added to the plates and incubated for 2 h at room temperature on a plate shaker adjusted to 700 rpm. After a 2 h incubation, plates were washed 3 times with a 1× wash buffer. MSD Sulfo-Tag anti-human IgG was added and incubated for 1 h at room temperature on a plate shaker at 700 rpm. Plates were subsequently washed 3 times and 150 uL/well of detection read buffer was added immediately prior to the plate read. Raw data were analyzed using GraphPad Prism software v10.1.2. Serum antibody titers or concentrations were calculated relative to the provided Reference Standard and denoted in arbitrary units (AU/mL).

### 2.5. Criteria for Defining Infection

Infection was defined as having any of the following three criteria: positive RBD-specific ELISA pre-vaccination [15] or non-vaccinated, high nucleocapsid antibody titers, or a person’s own statement of infection based on a positive COVID-19 test.

### 2.6. Quantification and Statistical Analysis

Cut-off values for all antibodies except for HCoV-OC43 and HCoV-HKU1 were calculated. Cut-off values were established at 3 standard deviations of the mean of all values of 29 pre-pandemic samples. Pearson correlation and non-parametric *t*-test analyses for cross-sectional antibody binding titers using the Mann–Whitney test were performed. The same donor antibody binding analyses were conducted using the Wilcoxon matched-pairs signed rank test. Antibody titers were log-transformed, and simple linear regression was used to calculate their R^2^ value. Statistical analysis was conducted using GraphPad Prism software v10.1.2.

## 3. Results

### 3.1. Demographics and Patient Characteristics

A total of 642 pHCWs were initially enrolled, of whom 337 participants returned for at least one follow-up visit after vaccination started in December 2020. No patients returned for follow-up while symptomatic from COVID-19 infection; therefore, there were no rescheduled appointments. The mean number of follow-up visits was 2 ± 1. Participants who returned for at least one follow-up visit were predominantly 31–40 years of age and female. In this cohort, 25% had comorbidities; however, 0% had any emergency department visit and/or hospitalization (Table 1).

### 3.2. Infection and Vaccination Rates

In this cohort, 301 (89%) participants were vaccinated, of whom 286 received the full primary vaccine series (2 doses), and 15 received one dose during the study period. Fifty-nine participants received an additional booster dose. Of those who were vaccinated, 54 were infected; a total of 33/54 were infected prior to receiving any vaccination, and 21/54 infections were breakthrough infections. Thirty-six participants were not vaccinated, of whom 22 (61%) were infected.

### 3.3. Analyses of SARS-CoV-2 IgG Antibody Response After Primary COVID-19 Vaccination

**Figure 2:**
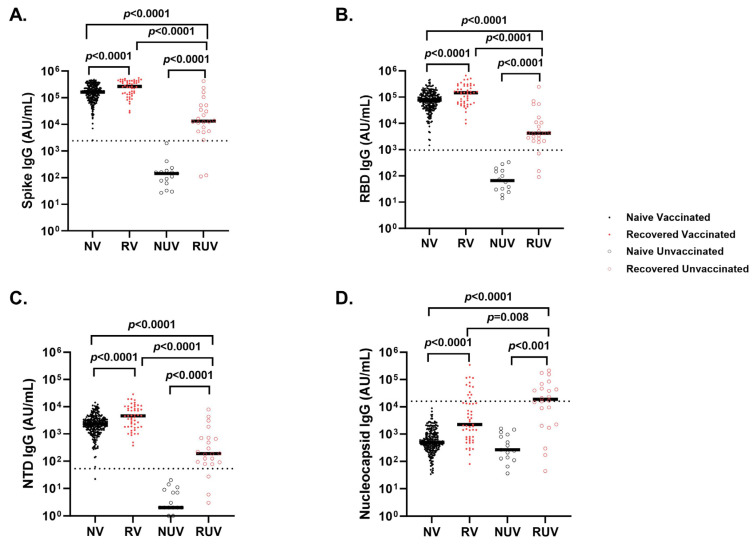
Antibody response to SARS-CoV-2 antigens across different subgroups of pediatric healthcare workers. IgG antibody response at first follow-up visit in *Naïve Vaccinated* (NV, vaccinated subjects without prior COVID-19 infection; closed black circle), *Recovered Vaccinated* (RV; those who recovered from prior COVID-19 infection; closed red circle), *Naïve Unvaccinated* (NUV; unvaccinated subjects never infected; open black circle) and *Recovered Unvaccinated* (RUV; unvaccinated subjects who have recovered from a prior COVID-19 infection; open red circle) against (**A**) Spike, (**B**) Receptor-Binding Domain (RBD), (**C**) N-terminal Domain (NTD), (**D**) Nucleocapsid. Vaccinated individuals show robust antibody binding titers for (**A**) RBD, (**B**) Spike, and (**C**) NTD compared to unvaccinated subjects. Recovered individuals show higher IgG antibody titers for (**A**) RBD, (**B**) Spike, and (**C**) NTD compared to naïve individuals regardless of vaccination status. (**D**) COVID-19-recovered subjects demonstrate higher nucleocapsid IgG antibody titers compared to naïve individuals irrespective of vaccination status. Nucleocapsid is a marker of infection; however, sampling post-infection was variable, and nucleocapsid titers dropped quickly. The dotted line in each figure corresponds to the specific cut-off value for each antibody. (**A**) Spike IgG = 2408 AU/mL; (**B**) RBD IgG = 962 AU/mL; (**C**) NTD = 53 AU/mL; (**D**) Nucleocapsid = 15,893 AU/mL.

**Figure 3:**
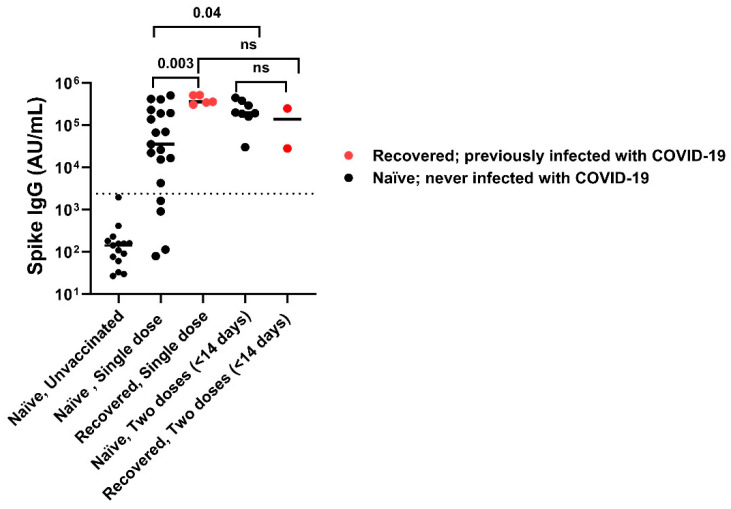
SARS-CoV-2 spike IgG titers with one dose versus two doses of vaccine. A single dose of vaccine is sufficient to attain maximum titers in recovered subjects compared to naïve subjects, while some naïve subjects required 2 doses of vaccination to attain maximum titer. After 2nd vaccination dose both naïve and recovered individuals achieved high Spike IgG antibody titers irrespective of infection. The dotted line corresponds to the cut-off value determined for spike IgG = 2408 AU/mL. ns: not significant.

Vaccinated individuals had robust antibody binding titers against RBD, NTD, and spike compared to non-vaccinated. Recovered vaccinated participants had higher titers compared to naïve vaccinated participants for Spike (Figure 2A) (278,950 ± 150,279 AU/mL vs. 181,337 ± 111,482 AU/mL; *p* < 0.0001), RBD (Figure 2B) (172,742 ± 141,903 AU/mL vs. 96,859 ± 75,468 AU/mL; *p* < 0.0001), NTD (Figure 2C) (6323 ± 5810 AU/mL vs. 2905 ± 2364 AU/mL; *p* < 0.0001), and nucleocapsid (Figure 2D) (23,323 ± 55,483 AU/mL vs. 878 ± 1071 AU/mL; *p* < 0.0001). Recovered individuals had higher titers than naïve individuals regardless of vaccination (*p* < 0.0001). Recovered unvaccinated participants had higher titers compared to naïve unvaccinated for Spike (55,335 ± 99,742 AU/mL vs. 256 ± 481 AU/mL; *p* < 0.0001), RBD (22,283 ± 52,599 AU/mL vs. 107 ± 1023 AU/mL; *p* < 0.0001), NTD (967 ± 1884 AU/mL vs. 6 ± 6 AU/mL; *p* < 0.0001), and nucleocapsid (44,411 ± 60,319 AU/mL vs. 537 ± 528 AU/mL; *p* < 0.0001). The highest titer response was observed in recovered vaccinated participants (Figure 2A–C). Nucleocapsid titers were significantly higher in recovered individuals with no titer response to vaccination (*p* < 0.0001) (Figure 2D). The single-dose vaccine elicited a statistically significant higher antibody response to Spike in recovered participants compared to naïve individuals (409,027 ± 98,502 AU/mL vs. 123,740 ± 161,425 AU/mL; *p* = 0.003), who needed both doses of vaccination to attain similar titers (Figure 3). After a second dose, both naïve and recovered individuals reach similar and high titers within <14 days.

### 3.4. Cross-Reactivity of Seasonal and Other Coronaviruses After COVID-19 Vaccination and Infection

**Figure 4:**
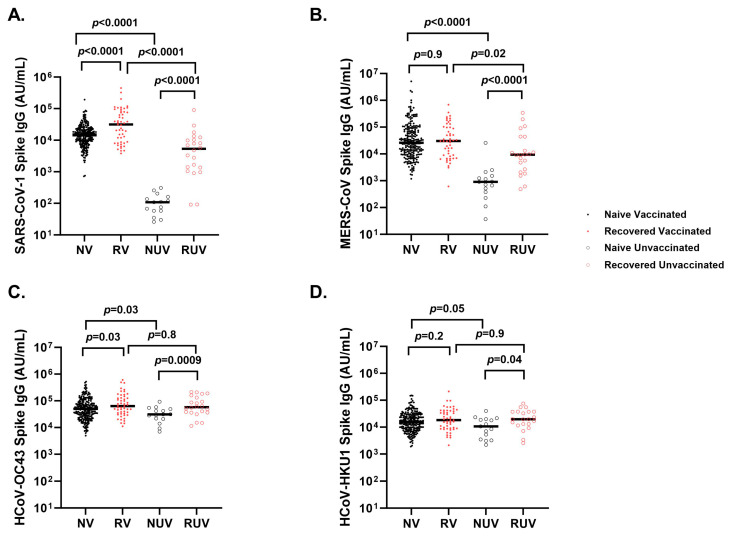
Spike IgG titers to seasonal and other coronaviruses in different subgroups of pediatric healthcare workers. Spike IgG antibody response at first follow-up visit in *Naïve Vaccinated* (NV, vaccinated subjects without prior COVID-19 infection; closed black circle), *Recovered Vaccinated* (RV; those who recovered from prior COVID-19 infection; closed red circle), *Naïve Unvaccinated* (NUV; unvaccinated subjects never infected; open black circle), and *Recovered Unvaccinated* (RUV; unvaccinated subjects who have recovered from a prior COVID-19 infection; closed red circle) against (**A**) SARS-CoV-1, (**B**) MERS-CoV, (C) HCoV-OC43, and (**D**) HCoV-HKU1. Vaccinated subjects show robust IgG titers for (**A**) SARS-CoV-1, and (**B**) MERS-CoV versus unvaccinated subjects. Recovered individuals had higher titers for (**A**) SARS-CoV-1 compared to naïve individuals regardless of vaccination status. (**C**) Spike IgG titers against HCoV-OC43 show a significant difference between naïve and recovered regardless of vaccination. (**D**) Spike IgG titers against HCoV-HKU1 differed between RUV and NUV (*p* = 0.04), as well as NV and NUV (*p* = 0.05).

Naïve vaccinated participants generate higher anti-spike titers compared to naïve unvaccinated participants for SARS-CoV-1 (Figure 4A) (18,823 ± 18,557 AU/mL vs. 117 ± 85 AU/mL; *p* < 0.0001), MERS-CoV (Figure 4B) (112,359 ± 398,473 AU/mL vs. 2593 ± 6484 AU/mL; *p* < 0.0001), HCoV-OC43 (Figure 4C) (71,063 ± 75,616 AU/mL vs. 35,347 ± 22,169 AU/mL; *p* = 0.03), and HCoV-HKU1 (Figure 4D) (22,323 ± 22,429 AU/mL vs. 13,146 ± 10,429 AU/mL; *p* = 0.05). Recovered unvaccinated participants had higher anti-spike titers compared to naïve unvaccinated participants for SARS-CoV-1 (10,451 ± 19,140 AU/mL vs. 117 ± 85 AU/mL; *p* < 0.0001), MERS-CoV (42,025 ± 81,909 AU/mL vs. 2593 ± 6484 AU/mL; *p* < 0.0001), HCoV-OC43 (84,051 ± 65,860 AU/mL vs. 35,347 ± 22,169 AU/mL; *p* = 0.0009), and HCoV-HKU1 (25,874 ± 19,134 AU/mL vs. 13,146 ± 10,429 AU/mL; *p* = 0.04). There was a strong correlation between SARS-CoV-2 anti-spike and SARS-CoV-1 anti-spike (*r* = 0.72, *p* < 0.0001) and a weaker correlation with MERS-CoV anti-spike (*r* = 0.13, *p* < 0.001). HCoV-HKU1 (*r* = 0.19, *p* < 0.0001) and HCoV-OC43 (*r* = 0.23, *p* < 0.0001).

### 3.5. Durability of SARS-CoV-2 Anti-Spike IgG Antibodies over Time After COVID-19 Primary Vaccination and Booster

**Figure 5:**
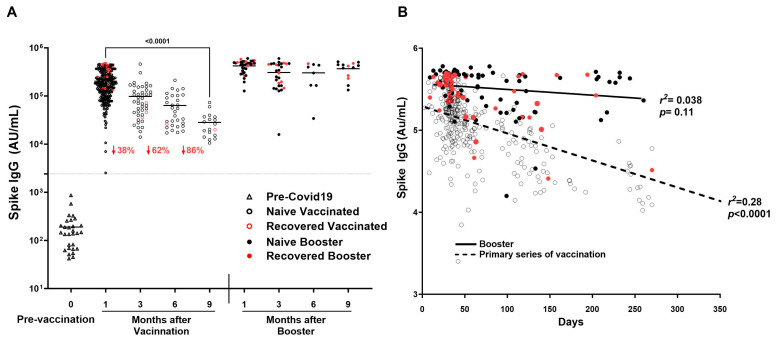
Durability of SARS-CoV-2 IgG antibodies over time after COVID-19 primary vaccination and booster. SARS-CoV-2 IgG antibody response was studied in naïve vaccinated (open black circle) and recovered vaccinated (open red circles) subjects at 1, 3, 6, and 9 months after the COVID-19 primary vaccination series (2 doses) and in booster recovered (subjects with breakthrough COVID-19 infection after receiving primary vaccine series and the COVID-19 booster) (red filled circle) and naïve booster subjects (never infected with COVID-19) (black filled circle) at 3, 6, and 9 months after COVID-19 booster. (**A**) Mean Spike IgG antibody titer to COVID-19 decreased by 86% by nine months after the primary series of COVID-19 vaccination, while Spike IgG antibody titers to COVID-19 showed sustained titer levels nine months after the COVID-19 booster. (**B**) A significant decline of Spike IgG antibody titer associated with an increase in time in days is observed in subjects after the COVID-19 primary series of vaccination (r^2^ = 0.28, *p* < 0.0001) absent in subjects receiving the COVID-19 booster (r^2^ = 0.038, *p* = 0.11). The dotted line corresponds to the cut-off value determined for spike IgG = 2408 AU/mL.

SARS-CoV-2 Spike IgG decreased by 86% by 9 months after the primary series of vaccination (Figure 5A), showing a significant decline of IgG as time passes in increasing days (*r*^2^ = 0.2773, *p* < 0.0001) (Figure 5B). In addition, titers decreased significantly between 1 and 3 months (190,243 ± 115,247 AU/mL vs. 96,195 ± 95,285 AU/mL; *p* < 0.0001) and between 1 and 6 months (190,243 ± 115,247 AU/mL vs. 68,814 ± 49,882; *p* < 0.0001). Booster showed consistency in titers up to 9 months as compared to the primary series of vaccination (*r*^2^ = 0.0384, *p* = 0.1101) (Figure 5A,B).

### 3.6. Analyses of SARS-CoV-2 Spike IgG Antibody Response to mRNA-1273 vs. BNT162b2 Vaccines

**Figure 6:**
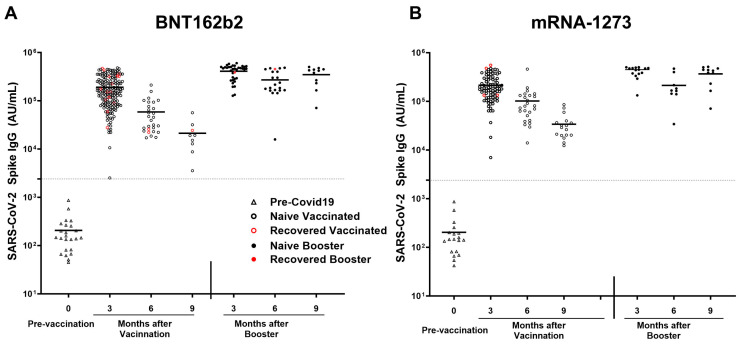
SARS-CoV-2 anti-spike IgG titers after mRNA-1273 and BNT162b2 vaccination and breakthrough infection. SARS-CoV-2 IgG antibody response was studied in naïve vaccinated (open black circle) and in recovered vaccinated (open red circle) subjects at 3, 6, and 9 months after the COVID-19 primary vaccination series and in naïve booster subjects (never infected with COVID-19; black filled circle) and booster recovered (subjects with breakthrough COVID-19 infection after receiving two doses of the primary COVID-19 vaccine and the COVID-19 booster; red filled circle) at 3, 6, and 9 months after COVID-19 booster who received (**A**) BNT162b2 and (**B**) mRNA-1273 vaccines. Breakthrough infection with COVID-19 was higher in subjects receiving the (**A**) BNT162b2 (n = 17 out of 203 vaccinations) as compared to subjects receiving the (**B**) mRNA-1273 vaccine (n = 4 out of 97 vaccinations), this was not statistically significant. The dotted line corresponds to the specific limit of detection for spike IgG = 2408 AU/mL.

All participants receiving either mRNA-1273 or BNT162b2 vaccine elicited a robust antibody response. In the naïve subgroup, participants receiving the mRNA-1273 vaccine (n = 85) elicited a higher response in antibodies compared to naïve participants receiving the BNT162b2 (n = 152) for Spike (209,343 ± 115,506 AU/mL vs. 165,676 ± 106,368 AU/mL; *p* = 0.003), RBD (116,469 ± 81,966 AU/mL vs. 85,892 ± 69,473 AU/mL; *p* = 0.0007), NTD (3564 ± 2349 AU/mL vs. 2467 ± 2103 AU/mL; *p* < 0.0001), and SARS-CoV-1 (22,460 ± 17,319 AU/mL vs. 15,669 ± 12,473 AU/mL; *p* = 0.0004). All participants receiving BNT162b2 demonstrated a larger decline in anti-spike antibody titers after the primary series of vaccination (*r*^2^ = 0.3694; *p* < 0.0001), vs. all participants receiving mRNA-1273(*r*^2^ = 0.2782; *p* < 0.0001). Breakthrough infection was higher among pHCWs who received BNT162b2 (n = 17; 8.4%) compared to mRNA-1273 (n = 4; 4.1%); however, it was not statistically significant (*p* = 0.2) (Figure 6A,B).

## 4. Discussion

In this prospective longitudinal study, we found that the IgG response to SARS-CoV-2 spike, NTD, and RBD antigens was strongest in individuals who had been infected prior to mRNA vaccination. This observation supports similar findings in the literature that demonstrate that previous COVID-19 infection enhances the antibody response to subsequent mRNA vaccination [16]. Unsurprisingly, antibodies against nucleocapsid were only elevated in recovered participants, as this viral protein is not present in the mRNA vaccine formulation. In addition, we found that one dose of vaccine triggered a robust immune response against spike protein in COVID-19 convalescent individuals, while naïve individuals may require two doses to achieve high antibody titers similar to previous studies [17,18,19]. This observation can help guide vaccination strategies in the setting of recent infection.

We have previously shown that participants infected with COVID-19 showed accelerated SARS-CoV-2 antibody decline, with undetectable titers in nearly half of participants at 9 months [13]. This finding is supported by similar results on pHCWs [20]. Although the presence of antibodies against RBD does not ensure immunity, we have also shown that anti-RBD antibodies correlated strongly with neutralization antibodies (r = 0.43, *p* < 0.0001). Since this correlation is established and published by previous studies as well [15,21], it suggests that a stronger binding antibody response will likely have more protection against COVID-19 infection.

The primary series of vaccinations generated a strong initial immune response, and anti-spike titers waned quickly over time in our study. However, titers remained above the cut-off value and were detectable at 9 months in contrast to infection alone. A similar decrease has been noted in another cohort of pHCWs receiving both the BNT162b2 and mRNA-1273 vaccines. Our study also suggests that anti-spike titers are more durable at 9 months post booster compared to the primary series of vaccinations. This observation was evident in individuals who received both BNT162b2 and mRNA-1273 vaccines, supporting prior work on booster-extended humoral half-lives compared to primary vaccination series in both HCWs and non-HCWs cohorts [22,23,24,25,26]. Recognition of the dose-dependent waning of antibodies is particularly important given that prior studies suggest some hesitancy among HCWs to receive booster vaccinations for COVID-19 [27,28,29,30,31].

Cross-reactivity between antibodies generated by COVID-19 infection or vaccination and other coronaviruses has also been a subject of significant interest. The MSD SARS-CoV-2 Panel 1 used in this study included spike proteins from SARS-CoV-2 (Wuhan strain), SARS-CoV-1, MERS, and the seasonal coronaviruses HCoV-OC43 and HCoV-HKU1. The latter two are widely circulating endemic viruses, while SARS-CoV-1 and MERS are epidemic viruses. SARS-CoV-1 and to a lesser degree MERS are somewhat homologous to SARS-CoV-2 spike at 79% and 50% genetic similarity, respectively [32]. SARS-CoV-1 was detected in China in 2002 [33] and infected about 8000 people, while the ongoing MERS epidemic was initially detected in 2012 [34], and is spread mostly in the Arabian Peninsula. Our cohort was unlikely to be exposed to SARS-CoV-1 and MERS-CoV. On the other hand, prior exposure to common cold coronaviruses is common in the general population, making them critical for understanding potential pre-existing cross-reactive immunity that may influence antibody response. Of note, the MSD assay employed here detected no signal to SARS-CoV-2, SARS-CoV-1, or MERS in pre-pandemic serum samples. In contrast, titers to the seasonal coronaviruses were readily detected.

In our cohort, we showed that antibodies generated after COVID-19 infection and vaccination may have cross-reactivity with the spike proteins of SARS-CoV-1 and MERS-CoV, with the strongest correlation to SARS-CoV-1, which is not surprising given their higher sequence homology. Additionally, we observed a correlation between antibody titers against SARS-CoV-2, HCoV-HKU1, and HCoV-OC43, suggesting cross-reactivity.

Beta-coronaviruses HCoV-OC43 and HCoV-HKU1 cause frequent mild childhood infections and provide transient immunity against other coronaviruses [35]. As such, we and others theorized that recent infection with mild coronaviruses could possibly provide some protection in children through coronavirus IgG cross-reactivity and potentially account for the mild symptomology of COVID-19 infection in this young population. The presence of cross-reactive anti-COVID-19 (Spike and RBD) antibodies in pre-pandemic samples from children and young adults that correlated with anti-HKU1 and anti-OC43 antibody titers has been documented [7]. In addition, children infected with COVID-19 showed higher antibody levels against HCoV-HKU1 and OC43 compared to seronegative children [9]. Cross-reactivity or limited cross-reactivity, with the absence of neutralization activity, has also been reported [8,36,37]. Within our cohort, pHCWs infected with COVID-19, regardless of vaccination status, had no hospitalizations or emergency visits, suggesting a milder course of infection. This finding contrasts with reports on mortality and morbidity of COVID-19 in non-pediatric HCWs, with a reported mortality rate ranging between 0.2 and 1.5%, and hospitalization rates up to 15.1% [38,39,40]. The trend of milder COVID-19 infection in our pHCWs cohort may, in part, be attributed to the frequent exposure pHCWs have with children in the workplace, potentially increasing contact with seasonal coronaviruses and providing partial immunity. Since this study did not explore associations between seasonal coronaviruses’ antibodies and neutralization, we cannot say with certainty that these antibodies offer protection from infection. However, we hypothesize that the presence of seasonal coronavirus antibodies may limit the severity and duration of symptoms, as suggested by Gouma and colleagues, who observed a significant negative correlation between seasonal coronavirus antibody titers and symptom duration in HCWs with COVID-19 [41]. In addition, a study by Asamoah-Boaheng and colleagues showed that HCWs vaccinated for COVID-19 generated higher HCoV-HKU1 and OC43 anti-spike antibodies compared to unvaccinated HCW [42], an observation also noted in this study.

Our results show that both BNT162b2 and mRNA-1273 vaccines elicited a robust antibody response against SARS-CoV-2 spike. Naïve participants receiving the mRNA-1273 vaccine mounted statistically significant higher antibody titers against spike, NTD, RBD, and SARS-CoV-1 spike antigens compared to BNT162b2. The mRNA-1273 vaccine delivers 100 µg of mRNA [43] that translates into the spike protein compared to 30 µg of mRNA delivered by the BNT162b2 vaccine [44], which likely contributes to the difference in titers. This difference in dosage could also account for the higher rate of breakthrough infections in participants who received BNT162b2 vaccines compared to those who received mRNA-1273 vaccines. Though statistically insignificant in our study, our finding supports the data in the literature [45,46]. However, while the mRNA-1273 vaccination may offer more protection, it is also associated with a higher frequency and severity of side effects [47,48]. We did not explore vaccine-specific side effect profiles in this study, and vaccine preferences ultimately depend on patient-specific needs, as well as vaccine availability.

This study has several limitations. This was a single-center study capturing a single professional category of pHCWs that is not generalizable to the public and may not be generalizable to non-pediatric HCWs. In addition, not all patients enrolled in the original cohort returned for follow-up. Although participants were scheduled for follow-up visits every three months, adherence to this schedule was inconsistent, with fewer pHCWs returning over time, leading to gaps in data collection that could impact the accuracy of our findings. Additionally, participants variably presented for antibody testing weeks to months after infection, a delay that likely resulted in lower antibody titers due to the natural waning of immune response over time. Therefore, the measured antibody levels reported may underestimate actual peak responses following infection. In addition, due to many mutations from the Wuhan strain in the RBD of the SARS-CoV-2 virus, antibodies from natural infections with subsequent variants, especially Omicron variants from December 2021 onward to completion of the study in May 2022, may not have worked well with this Wuhan RBD spike antigen in the MSD test. However, we also observed that vaccinated and/or previously infected individuals demonstrated robust responses across multiple SARS-CoV-2 antigens, including NTD and spike. These factors should be considered when interpreting our results, as they may have influenced the observed trends in antibody titers and their correlations with infection and immunity.

This longitudinal study also has several notable strengths. The analysis of IgG titers against a comprehensive array of antigens, including SARS-CoV-2 anti-spike, RBD, NTD, and nucleocapsid, as well as anti-spike of other coronaviruses provides critical insights into cross-reactivity and immunity across coronaviruses. The longitudinal design enables an in-depth understanding of immune durability and the dynamic changes in the antibody response over time. Additionally, the examination of cross-reactivity provides insights that may inform future pandemic preparedness. Collectively, these strengths highlight the study’s significant implications for public health and vaccination regimens.

## 5. Conclusions

As COVID-19 has become endemic, antibodies against SARS-CoV-2 are useful in understanding and estimating the period of protection provided by either infection or vaccination. The rise in and duration of antibody titers against COVID-19 are also important in understanding the efficacy of the mRNA vaccines and guiding future vaccination regimens. This study found that 100% of pHCWs mounted significant antibody titer responses to SARS-CoV-2 with vaccination. While titers waned quickly after the primary vaccination series, the duration of antibody titers was maintained after a COVID-19 booster. Longitudinal profiling of the immune response to COVID-19 vaccination and infection is crucial for understanding the durability and effectiveness of immunity over time. This research provides valuable insights into the dynamics of immune protection, particularly among pediatric healthcare workers who face regular exposure to infectious risks. By identifying patterns of immune waning and variability, these studies inform evidence-based public health policies, including vaccine scheduling, booster recommendations, and prioritization strategies for frontline workers. Furthermore, the findings guide the development of clinical guidelines for managing breakthrough infections, customizing immunization approaches, optimizing resource allocation, enhancing pandemic preparedness, and ultimately strengthening the overall impact of vaccination programs.

## Figures and Tables

**Table 1:** Demographics and characteristics of pediatric healthcare workers enrolled in a longitudinal study exploring antibody dynamics in infection and vaccination.

Characteristic	All (n = 337)
**Median Age Range (years)**	31–40
**Gender-Female n (%)**	286 (84%)
**Vaccinated** n (% of ALL)	301 (89%)
BNT162b2 n (% of vaccinated)	203 (67%)
mRNA-1273 n (% of vaccinated)	97 (32%)
Ad26.COV2.S n (% of vaccinated)	1 (1%)
**Booster ^a^-Yes n (%)**	59 (18%)
**Infected ^b^-Yes n (%)**	76 (23%)
**Comorbidities ^c^-Yes n (%)**	85 (25%)
**Emergency Department Visit-Yes n (%)**	0 (0%)
**Hospitalization-Yes n (%)**	0 (0%)

^a^ Patients receiving third dose of vaccine after receiving primary series of vaccination (2 doses). ^b^ See methods for criteria defining infection. ^c^ Participants were asked the following question “Do you have a pre-existing condition? (Yes/No)”.

## Data Availability

Data is contained within the article.

## Abbreviations

The following abbreviations are used in this manuscript:

SARS-CoV-2	Severe Acute Respiratory Syndrome Coronavirus 2
HCWs	Healthcare workers
pHCWs	Pediatric healthcare workers
PPE	Personal Protective Equipment
IgG	Immunoglobulin G
RBD	Receptor Binding Domain
NTD	N-terminal domain
MERS-CoV	Middle East Respiratory Syndrome Coronavirus
SARS-CoV-1	Severe Acute Respiratory Syndrome Coronavirus 1
HCoV-HKU1	Human coronavirus HKU1
HCoV-OC43	Human coronavirus OC43
